# Use of wide-field optical fluorescence for visualization of oral biofilm in a patient with peri-implant mucositis: a new approach

**DOI:** 10.31744/einstein_journal/2021RC5638

**Published:** 2021-05-13

**Authors:** Sérgio Araújo Andrade, Sebastião Pratavieira, Vanderlei Salvador Bagnato, Fernando de Pilla Varotti

**Affiliations:** 1 Universidade Federal da São João del-Rei DivinópolisMG Brazil Universidade Federal da São João del-Rei, Divinópolis, MG, Brazil.; 2 Universidade de São Paulo São CarlosSP Brazil Universidade de São Paulo, São Carlos, SP, Brazil.

**Keywords:** Biofilms, Dental implantation, Diagnosis, oral, *Gram*-negative anaerobic bacteria, Mucositis

## Abstract

Peri-implant diseases, caused by bacteria from biofilm related to dental implants, are one of the main causes of late loss of implants. In this sense, peri-implant diseases are divided into peri-implant mucositis, when it affects only the soft tissues, and peri-implantitis, when there is a bone involvement, which can lead to the failure of dental implant therapy. Thus, biofilm removal is essential for peri-implant health, allowing long-term success in implant therapy. To improve the visualization of oral biofilm, which is usually transparent or colorless, disclosing agents have been routinely used. However, disclosing agents have allergenic potential and can cause staining extrinsically in restorative and prosthetic materials, leading to aesthetic impairment. Thus, the use of fluorescence has been studied as an alternative for visualization of oral biofilm. Therefore, this report describes the use of wide-field optical fluorescence for visualization of oral biofilm associated with implants and teeth, in a routine appointment and follow-up of a partially edentulous patient with peri-implant mucositis. In addition, this report showed wide-field optical fluorescence can be used in a clinical routine of care of patients with dental implants. In this sense, wide-field optical fluorescence allowed easy and immediate visualization of the mature oral biofilm for its adequate removal, evaluation of the quality of restoration to sealing of screw access-hole of implant and identification of cariogenic lesions, without risk of allergic reactions or staining of prostheses and restorations.

## INTRODUCTION

Nowadays, there is an exponential growth in the number of patients, either partially or totally edentulous, successfully rehabilitated through dental implants.^(^^1^^–^^3^^)^ However, despite the constant scientific progress in all areas concerning dental implants, there is still a late loss of implants related to peri-implant diseases, caused by bacteria from biofilm related to dental implants.^(^^2^^–^^5^^)^ In this sense, peri-implant diseases are divided into peri-implant mucositis, when it affects only the soft tissues, and peri-implantitis, when there is a bone involvement, which, can lead to the failure of the therapy with dental implants.^(^^2^^,^^4^^,^^6^^)^ Thus, biofilm removal is essential for periodontal and peri-implant health allowing long-term success in implant therapy.^(^^4^^,^^7^^,^^8^^)^

Since, biofilm formation around implants and teeth has been established as similar, this also suggests that there is similarity in the clinical characteristics and bacteria involved in periodontal and peri- implant disease.^(^^9^^,^^10^^)^ Thus, the oral biofilm formation process begins with adhesion of the bacteria to the acquired pellicle on the teeth, implants or prosthetic components. Over time, oral biofilm accumulates and becomes more complex with dominant presence of *Gram*-negative anaerobic bacteria, such as *Porphyromonas gingivalis, Treponema denticola, Tannerella forsythia, Prevotella intermedia, Fusobacterium nucleatum, Aggregatibacter actinomycetemcomitans* and *Eikenella corrodens.*^(^^10^^,^^11^^)^ In this sense, these *Gram*-negative anaerobic bacteria are the main pathogens related to both periodontal disease as well as peri-implant disease.^(^^7^^,^^12^^,^^13^^)^ Subsequently, if there is no removal of this mature biofilm, it can calcify, sub- or supragengivally, resulting in the calculus.^(^^11^^)^ Thereby, it is evident the need to evaluate the implants and their prosthetic components for the presence of mature biofilm and calculus, which must be removed to prevent peri- implant diseases.^(^^9^^)^

Oral biofilm is usually transparent or colorless, which makes it difficult to visualize it for subsequent removal.^(^^11^^,^^14^^,^^15^^)^ Thus, to allow visualization of oral biofilm was developed, disclosing agent, which is a traditional method for staining of biofilm.^(^^8^^,^^11^^,^^14^^)^ However, disclosing agents may cause staining extrinsically in restorative and prosthetic materials, such as acrylic resin and composite resins and resin-modified glass ionomer, causing aesthetic impairment and patient dissatisfaction.^(^^16^^,^^17^^)^ Therefore, since these materials are often used in prosthesis over implant, or in screw access-hole restorations, the use of disclosing agents is a problem. In addition, disclosing agents have been described as potentially allergenic.^(^^14^^)^

The use of fluorescence has been widely studied for visualization of oral biofilm related to periodontal disease and decays.^(^^11^^–^^13^^,^^18^^)^ In this sense, quantitative light-induced fluorescence technique (QLF), which uses 405nm wavelength light for illumination of the oral cavity, permits the observation of mature oral biofilm, with orange or reddish coloration, due to the presence of porphyrins produced by certain *Gram*-negative anaerobic bacteria.^(^^8^^,^^15^^,^^19^^–^^21^^)^ However, despite the correlation between mature pathogenic biofilm and reddish fluorescence visualization, the use of QLF in the clinical routine still is not cost-effective. Otherwise, wide-field optical fluorescence (WOF) visualization technique has a better cost-effectiveness ratio for use in clinical routine, and applies the same principles of QLF due to the use of light in the same wavelength range. Nevertheless, until then, almost all studies on WOF were related to the detection of potentially malignant oral lesions or oral cancer.^(^^22^^–^^24^^)^ Recently, we have demonstrated the use of wide-field fluorescence in the clinical routine in different areas of dentistry and medicine.^(^^25^^–^^27^^)^ However, until then, there were no reports in the literature about the use of WOF in clinical routine, for visualization of oral biofilm associated with the implants.

The present report describes the use of WOF for visualization of oral biofilm associated with implants and teeth in a routine consultation of a partially edentulous patient rehabilitated with implants and presenting peri-implant mucositis.

The care protocol consisted of conventional clinical examination, WOF examination and, then mechanically cleaning, polishing, and oral hygiene instructions to the patient. An intraoral camera model DP6 Scope^®^ (RF System Lab., Almere, Netherlands), coupled to a computer, was used to capture clinical images. Evince^®^ device (MMOptics, São Carlos, Brazil), with light source emitting at 400±10nm was used to carry out the WOF, whereas a digital microscope model Deluxe Hand- held Digital Microscope^®^ (Celestron LLC, Torrance, CA, USA) was used coupled to Evince^®^ and to a computer to obtain the optical fluorescence imaging.

The mechanically cleaning and polishing procedures for removal of oral biofilm and calculus in region of implants were performed using cups and silicone cones (Microdont, São Paulo, SP, Brazil) with polishing paste (Clinpro™ Prophy Paste, 3M Oral Care, Saint Paul, MN, USA) and kit for implant scaling (Implacare™, Hu-Friedy Mfg. Co., Chicago, IL, USA). In the dental region, ultrasound (PROFI II Ceramic, Dabi Atlante, Ribeirão Preto, SP, Brazil) with metal tips (Dabi Atlante, Ribeirão Preto, SP, Brazil) and metal scalers (Hu-Friedy Mfg. Co., Chicago, IL, USA) were employed. In addition, the same type of polishing paste, cups and silicone cones used in implants were used on the teeth.

Oral hygiene instructions were given to the patient, presenting the reddish fluorescence images of oral biofilm, so that the patient could identify regions with highest biofilm accumulation and remove it. The care protocol was performed on the day of initial consultation, and repeated after 15 days. Thirty days after the first appointment, only a new clinical examination and wide-field fluorescence were performed.

This study was approved by the Ethics Committee of *Universidade Federal de São João del-Rei, Campus Centro-Oeste Dona Lindu* (approval number: 1.756.617; CAAE: 59621516.8.0000.5545). In addition, it is stated that the patient signed the Informed Consent Form.

## CASE REPORT

A 55-year-old female patient, partially edentulous and rehabilitated with prostheses over implants, attended the *Centro de Diagnóstico Oral do Departamento de Saúde de Divinópolis* at Minas Gerais, Brazil, for routine consultation, complaining of swelling and bleeding in peri-implant mucosa of implants, in region of 12 and 11 teeth, perceived 3 months before. The patient reported diabetes, history of various surgeries due to periodontal disease in posterior teeth, having been submitted to oral rehabilitation by means of implants, 8 years before, with another professional, and no current complaint of pain.

### Clinical observations

**Figure 1:**
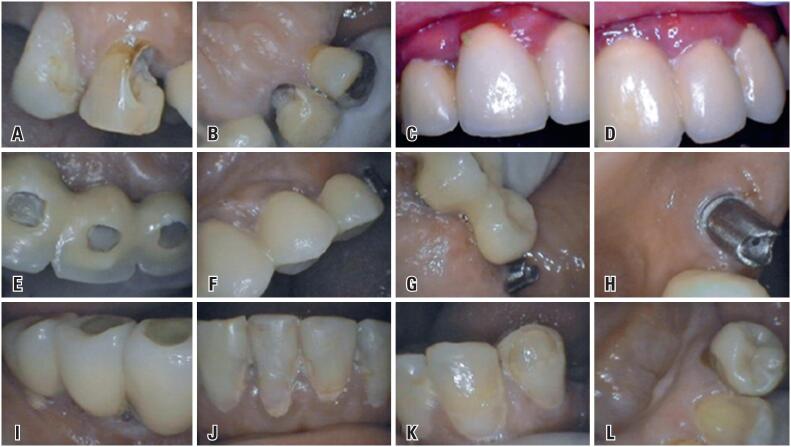
Initial clinical images. Carious lesion on tooth 13 on the mesiobuccal (A) and palatal surfaces (B); Edema of the peri-implant mucosa in the region of 12 to 23, with dental calculus in 12 and bleeding in 12 and 11 (C and D); Screw access-hole restorations of implants in 12,11 and 21 (E); Prostheses over implants from 23 to 25, and erythema of the mucosa is observed in 25 (F); Palatal region from 24 to 26, in which erythema is observed in the mesial of 24 and, prosthetic component of the implant in the region of 26, with loss of the prosthetic crown (G and H); Buccal view of the implants and their screw access-hole restorations from 44 to 46 (I); Teeth 32 to 42 with unfinished and unpolished restorations (J); Buccal view of teeth 33 and 34 (K) and lingual (L), and 34 has prosthetic restoration

The presence of carious lesion was verified in tooth 13, in the mesial of the buccal and palatine surfaces (Figures 1A and 1B), edematous and erythematous area in the peri-implant mucosa from 12 to 23, associated with dental calculus in 12, and bleeding in 12 and 11 (Figures 1C and 1D). In turn, the resin restorations in region of screw access-hole of implants in 12,11 and 21 were properly sealed (Figure 1E). Erythematous regions were observed in mucosa in the buccal region of the 25, and in the interproximal region of the palatal surface of prosthesis, over implants between 23 and 24 (Figures 1F and 1G). In addition, the absence of prosthetic crown associated with implant in 26 was found (Figures 1G and 1H). The lower arch, in turn, had prostheses over implants from 44 to 46 and, teeth from 34 to 43 with extensive restorations, without finishing and polishing (Figures 1I, 1J, 1K and 1L). Furthermore, the patient had edentulous areas without rehabilitation, from 15 to 18, 27, 28, from 38 to 35, 47 and 48.

In probing, teeth presented values ≤2mm, except for the 14, which had a distal probing of 3mm, with no evidence of bleeding or suppuration but with slight mobility. In turn, implants presented values of probing ≤3mm, with presence of bleeding only in 12 and 11.

### Wide-field optical fluorescence visualization

**Figure 2:**
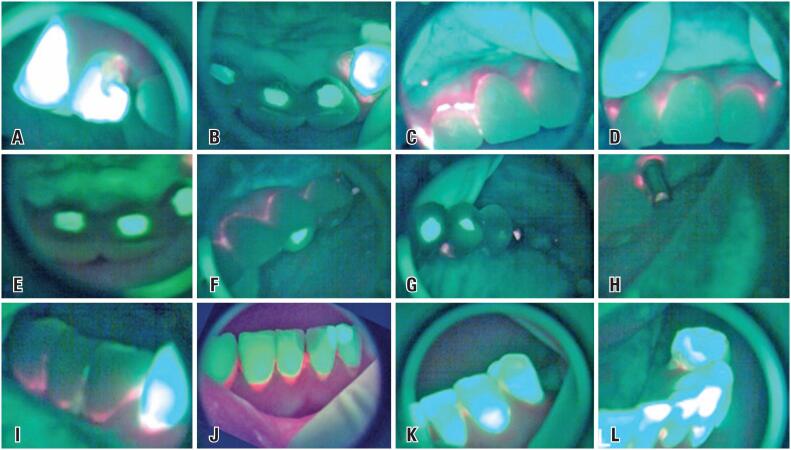
Initial images of wide-field optical fluorescence. Vestibular view of the region of 14 and 13 showing reddish fluorescence in the mesiobuccal region of 13 (A); Palatal view of the region of 13 to 21 showing reddish fluorescence in 13 and areas of intense green fluorescence in the region of screw access-hole restorations of implants 12, 11, and 21 (B); Vestibular view of the prostheses over implants from 12 to 22 showing reddish fluorescence in cervical, which is more intense in 12 and 11 (C and D); Palatal view with intense green fluorescence in region of the screw access-hole restorations of implants from 12 to 21 (E); Presence of reddish fluorescence in cervical of the buccal face of prostheses over implants from 23 to 25 (F); Palatal interproximal between 23, 24, cervical face and, in the access to the 26 prosthetic component screw (G and H); Intense green fluorescence of screw access-hole restorations of implants at 23 and 24 (F and G); Reddish fluorescence in cervical region of prostheses over implants from 46 to 44 (I); Cervical face of teeth from 43 to 31(I and J); Interproximal between the teeth 32 to 34 in buccal (K) and, in cervical of lingual face of the 34 (L)

Reddish fluorescence could be seen in the region of carious lesion of the 13 in vestibular and palatal surfaces (Figures 2A and 2B) and along the cervical of the vestibular surface of prostheses over implants from 12 to 21, which was more intense in 12 and 11 (Figures 2C and 2D). Intense green fluorescence was noted in region of screw access-hole restorations of implants from 12 to 21 (Figure 2E). In turn, reddish fluorescence was observed in the cervical of the buccal surface from 23 to 26, screw access-hole of implant in 26 and, in the interproximal palatal region between 23 and 24 (Figures 2F, 2G and 2H). The lower arch showed reddish fluorescence in cervical region of the prostheses over implants from 44 to 46, cervical of the teeth from 43 to 31, interproximal between the teeth from 32 to 34 and, in cervical of the lingual face of the 34 (Figures 2I, 2J, 2K and 2L).

### Radiographic examination

**Figure 3:**
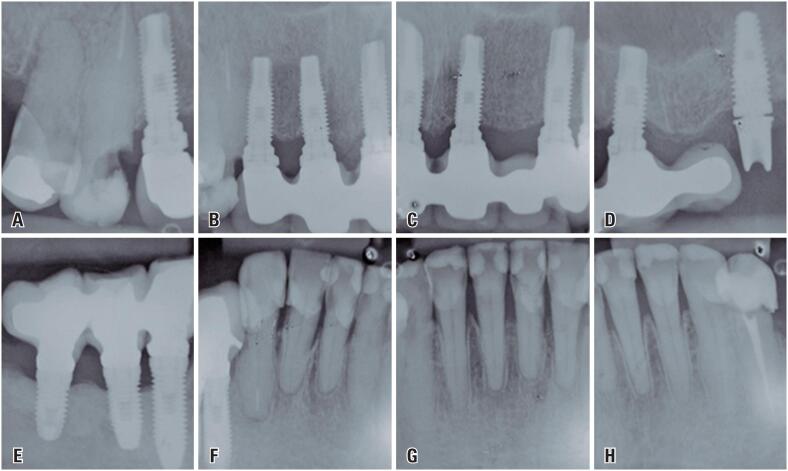
Initial radiographic images. Presence of prosthesis over implant in region of 12, bone loss in distal of tooth 14 and, radiolucent area in mesial of tooth 13, which was endodontically treated (A); Presence of prostheses over implants from 12 to 25, and 44 to 46, with normal bone pattern (B, C, D and E); Implant associated with its prosthetic component in region of 26, but with loss of the prosthetic crown (D); Teeth from 34 to 43, extensively restored (F, G and H), and 34 was endodontically treated and restored with prosthetic crown (H)

Periapical radiography showed bone loss in the distal of tooth 14 and a carious lesion in the mesial of tooth 13, which was endodontically treated (Figure 3A). Dental implants were present in the region of 12, 11, 21, 23, 24, 26, 44, 45 and 46 with images within the normal bone pattern (Figures 3A to 3E). There are prostheses over implants from 12 to 25, and 46, 45 and 44, being that in 26 there was no prosthetic crown related to the implant (Figures 3A to 3E). In turn, figures 3F to 3H showed bone loss ranging from mild to moderate, related to teeth from 33 to 43, and presence of widely restored teeth from 34 to 43, and tooth 34 was endodontically treated.

### Correlations between consultations: initial and follow-up with 15 and 30 days

**Figure 4:**
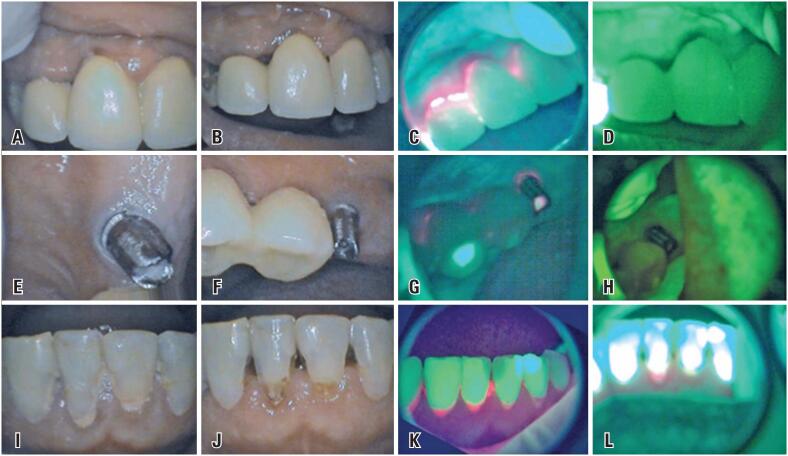
Clinical and fluorescence images referring to consultations: initial, follow-up with 15 and 30 days. Reduction of edema and absence of bleeding from the peri-implant mucosa in 12 and 11, 15 days after the initial consultation (A); After 30 days of the initial consultation, the region had normal characteristics (B); Presence of reddish fluorescence in cervical of prostheses over implants from 12 to 21, which was more intense in 12 and 11 at the initial consultation (C); After 30 days of the initial consultation, the same region showed an absence of reddish fluorescence (D); Peri-implant mucosa in region of the 26 with 15 days (E); After 30 days of the initial consultation, with normal characteristics (F); Initial aspect of reddish fluorescence around the prosthetic component and in screw access-hole of implant 26 (G); After 30 days of the initial consultation, the same region, showed absence of reddish fluorescence (H); Teeth from 32 to 42 with unfinished and unpolished restorations after 15 days (I); After 30 days of the initial consultation, restorations were properly finished, polished and there was the presence of a carious cervical lesion in 41 (J); Presence in the initial consultation of reddish fluorescence in cervical from 31 to 43 and interproximal between 31 and 41 (K); After 30 days, there was reddish fluorescence only in the cervical of the 41 (L)

The process of recovery of peri-implant mucosa health of 12 and 11 was noted by reduction of edema and absence of bleeding 15 days after the initial consultation (Figure 4A), whereas, after 30 days, peri-implant mucosa was in normal condition (Figure 4B). In this sense, in initial consultation, the cervical region of 11 and 12 showed presence of intense reddish fluorescence (Figure 4C), which, after 30 days, was absent (Figure 4D). The region of the implant corresponding to 26, in which there was no prosthetic crown, showed characteristics of normal mucosa at 15 days (Figure 4E) and 30 days (Figure 4F). In fact, in initial consultation, there was reddish fluorescence around the prosthetic component and at the screw access-hole of the 26 (Figure 4G), which, after 30 days, was absent (Figure 4H). Otherwise, initial presence of unfinished and non-polished restorations in region from 32 to 42 (Figure 4I), which, after 30 days, duly finished, polished and with biofilm removal allowed to identify carious cervical lesion in tooth 41 (Figure 4J). However, in this case, initial reddish fluorescence in cervical region of 31, 41, 42, 43 (Figure 4K) was still maintained after 30 days, only in the region of the cervical carious lesion of tooth 41 (Figure 4L).

A direct correlation between the presence of reddish fluorescence and peri-implant mucosa involvement was observed. Moreover, the area with the worst clinical picture, with bleeding between 12 and 11, corresponded to the area with the highest intensity of reddish fluorescence. It was evident that after adequate, calculus and oral biofilm removal, guided by previous identification of reddish fluorescence and accompanied by oral hygiene orientation to the patient, there was a complete reestablishment of peri-implant health in 30 days. The WOF exam also allowed it to be possible to assess the integrity of the sealing provided by the restorations of the screw access of the implants due to the contrast between the component material of the prosthesis and the restorative material.

The diagnosis was established as peri-implant mucositis due to the involvement of only the peri-implant mucosa tissue, painless, without bone loss or suppuration and without mobility in any implant. Thus, the patient was informed of the correlation between accumulation of oral biofilm and peri-implant mucositis and, therefore, the need to maintain strict oral hygiene especially in areas that showed reddish fluorescence. It was also explained to the patient about the importance of maintaining control of diabetes, not only to prevent a worsening of your oral health, but also maintain an adequate overall health. Subsequently, the patient was referred to another dentist for periodontal, restorative and prosthetic procedures, and periodontal and oral hygiene follow-up.

## DISCUSSION

Initially, it is noteworthy that the device used in the present case works with light emission at 400±10nm. Therefore, it benefits from the correlations obtained in studies carried out with either 400 or 405nm, since they share the same biophotonics characteristics. Thus, initial presence of reddish fluorescence in tooth 13 and, after 30 days of initial consultation, in the region of cervical lesion in tooth 41 is corroborated by the findings of König et al.,^(^^28^^)^ indicating that the most suitable wavelength for excitation and visualization of reddish fluorescence in carious lesions is 400nm.^(^^28^^)^ Otherwise, cervical lesion of tooth 31 probably has a non-carious origin as a loss of restorative material, thus, due to the absence of bacterial contamination the reddish fluorescence was absent.

However, this case can be considered well representative of possibilities of using WOF in follow-up of patients rehabilitated by implants, because the patient was partially edentulous and rehabilitated with implants, which illustrates well particularities that apply to the teeth and implants. In this sense, Lee et al.,^(^^10^^)^ Quirynen et al.,^(^^3^^)^ Kurtzman et al.,^(^^9^^)^ and Todescan et al.,^(^^6^^)^ emphasized that special care should be taken with partially edentulous patients, because the remaining teeth function as reservoirs of bacteria for implant colonization.^(^^3^^,^^6^^,^^9^^,^^10^^)^

Studies conducted with QLF by Coulthwaite et al.,^(^^19^^)^ Volgenant et al.,^(^^20^^)^ Han et al.,^(^^21^^)^ Kim et al.,^(^^15^^)^ Pretty et al.,^(^^11^^)^ and Lee et al.,^(^^8^^)^ have shown that only mature biofilm showed fluorescence in reddish or orange tones, which, is possibly due to anaerobic *Gram*-negative bacteria.^(^^8^^,^^11^^,^^15^^,^^19^^–^^21^^)^ In addition, Han et al.,^(^^21^^)^ emphasized the correlation between reddish fluorescence and presence of *Porphyromonas gingivalis, Prevotella intermedia* and *Fusobacterium nucleatum*, which are pathogenic anaerobic bacteria present in mature biofilm and related to periodontal disease.^(^^21^^)^ Bjurshammar et al.,^(^^29^^)^ showed that *Aggregatibacter actinomycetemcomitans* is capable of emitting reddish fluorescence and that there is correlation between colony growth time and increased reddish fluorescence intensity.^(^^29^^)^ Thus, these previous studies validate the use presented in this case for the WOF, to visualize mature biofilm of greater pathogenic potential, allowing its complete removal. Furthermore, report of the patient about the progression of 3 months for perception of the signs of peri-implant mucositis is more than enough to establish a mature and pathogenic biofilm. Likewise, removal of oral biofilm and maintenance of adequate oral hygiene, prevented mature biofilm formation and allowed recovery of peri-implant mucosa health, which showed correlation with absence of reddish fluorescence after 30 days of initial consultation.

The verifying of sealing provided by restorations of accesses to the screws of implants is of fundamental importance to avoid that, in case of a failure, allow the retention or penetration of biofilm in these areas. In this sense, Heinrich-Weltzien et al.,^(^^18^^)^ pointed out that the presence of reddish fluorescence may indicate penetration of the oral biofilm into the filling, microleakage, gap or partial loss of the filling.^(^^18^^)^ Thus, in this case report, a contrast between restorative material with bright light green fluorescence and the material of the prosthesis, with lower fluorescence, was readily verified through WOF. Therefore, restorations to sealing of screws access-hole were considered adequate due the non-visualization of reddish fluorescence.

Heinrich-Weltzien et al.,^(^^18^^)^ reported that fluorescence allows the practice of removing oral biofilm through the guidance of specific sites, and also allows to the professional to ensure that all biofilm has been removed after professional cleaning.^(^^18^^)^ Likewise, in this case report, the use of widefield fluorescence allowed the visualization of the mature oral biofilm in reddish tones, which served to direct both the professional and the patient as to which areas should receive more considerable attention in oral biofilm removal.

## CONCLUSION

The wide-field optical fluorescence can be used within a clinical routine of care of patients with dental implants. It allowed easy and immediate visualization of the mature oral biofilm for its adequate removal, evaluation of the quality of restoration to sealing of screw access-hole, and identification of caries in partially edentulous patients, with no risk of allergic reactions or staining of prostheses and restorations.
