# Supplementary material for: Use of wide-field optical fluorescence for visualization of oral biofilm in a patient with peri-implant mucositis: a new approach
Source: Einstein (Sao Paulo). 2021 May 13;19:eRC5638. doi: 10.31744/einstein_journal/2021RC5638 (PMC8121375; doi:10.31744/einstein_journal/2021RC5638)
Supplement: Translation

# Translation: Uso da fluorescência óptica de campo amplo para visualização de biofilme oral em paciente com mucosite peri-implantar: uma nova abordagem

**Authors:** Sérgio Araújo Andrade, Sebastião Pratavieira, Vanderlei Salvador Bagnato, Fernando de Pilla Varotti

**Affiliations:** 1 DivinópolisMG Brasil Universidade Federal da São João del-Rei, Divinópolis, MG, Brasil.; 2 São CarlosSP Brasil Universidade de São Paulo, São Carlos, SP, Brasil.

**Keywords:** Biofilmes, Implantação dentária, Diagnóstico bucal, Bactérias anaeróbias *Gram*-negativas, Mucosite

## Abstract

Doenças peri-implantares, causadas por bactérias de biofilme relacionadas a implantes dentários, são uma das principais causas de perda tardia de implantes. Nesse sentido, as doenças peri-implantares são divididas em mucosite peri-implantar, quando afeta apenas tecidos moles, e peri-implantite, quando há comprometimento ósseo, o que pode levar ao fracasso da terapia com implantes dentários. Assim, a remoção do biofilme é essencial para a saúde peri-implantar, permitindo sucesso a longo prazo na terapia com implantes. A fim de melhorar a visualização do biofilme oral, que geralmente é transparente ou incolor, agentes reveladores têm sido rotineiramente utilizados. No entanto, esses agentes têm potencial alergênico e podem causar manchas extrinsecamente em materiais restauradores e protéticos, levando a prejuízo estético. Assim, o uso da fluorescência tem sido estudado como alternativa para visualização do biofilme oral. Este relato descreve o uso da fluorescência óptica de campo amplo para visualização do biofilme oral associado a implantes e dentes em uma consulta de acompanhamento de rotina de uma paciente parcialmente edêntula com mucosite peri-implantar. Além disso, este relato evidenciou que a fluorescência óptica de campo amplo pode ser utilizada dentro da rotina clínica de atendimento de pacientes com implantes dentários. Nesse sentido, a fluorescência óptica de campo amplo permitiu a visualização fácil e imediata do biofilme oral maduro para sua remoção adequada, a avaliação da qualidade da restauração do selamento do orifício de acesso do parafuso do implante e a identificação de lesões cariogênicas, sem risco de reações alérgicas ou manchamento de próteses e restaurações.

## INTRODUÇÃO

Hoje em dia, há um crescimento exponencial no número de pacientes, quer parcial ou totalmente edêntulos, reabilitados com sucesso por meio de implantes dentários.^(^^1^^–^^3^^)^ Entretanto, a despeito do constante progresso científico em todas as áreas ligadas a implantes dentários, ainda há uma perda tardia de implantes relacionados a doenças peri-implantares, causadas por bactérias do biofilme relacionado a implantes dentários.^(^^2^^–^^5^^)^ Nesse sentido, as doenças peri-implantares estão divididas em mucosites peri-implantares, quando afetam apenas os tecidos moles, e peri-implantite, quando há envolvimento ósseo, o que pode levar à falha de terapia com implantes dentários.^(^^2^^,^^4^^,^^6^^)^ Assim, a remoção de biofilme é essencial para a saúde periodontal e peri-implantar, permitindo sucesso de longo prazo na terapia por implantes.^(^^4^^,^^7^^,^^8^^)^

Sendo que a formação de biofilme ao redor de implantes e dentes já foi estabelecida como similar, isso também sugere que há similaridade nas características clínicas e nas bactérias envolvidas nas doenças periodontais e peri-implantares.^(^^9^^,^^10^^)^ Por conseguinte, o processo de formação do biofilme oral se inicia com a adesão da bactéria à película adquirida nos dentes, implantes, ou componentes protéticos. Com o tempo, o biofilme oral se acumula e se torna mais complexo, com a presença dominante de bactérias anaeróbicas *Gram*-negativas, como *Porphyromonas gingivalis, Treponema denticola, Tannerella forsythia, Prevotella intermedia, Fusobacterium nucleatum, Aggregatibacter actinomycetemcomitans* e *Eikenella corrodens.*^(^^10^^,^^11^^)^ Nesse sentido, esses *Gram*-negativos são os principais patógenos relacionados tanto à doença periodontal quanto à doença peri-implantar.^(^^7^^,^^12^^,^^13^^)^ Subsequentemente, se não houver remoção desse biofilme maduro, ele pode se calcificar em situação sub- ou supragengival, resultando em cálculo.^(^^11^^)^ Com isso, é evidente a necessidade de avaliar os implantes e seus componentes protéticos quanto à presença de biofilme maduro e cálculos, que têm de ser removidos para prevenir doenças peri-implantares.^(^^9^^)^

O biofilme oral geralmente é transparente ou sem cor, o que torna sua visualização e subsequente remoção difíceis.^(^^11^^,^^14^^,^^15^^)^ Assim, para permitir a visualização do biofilme oral, foi desenvolvido um agente evidenciador, que é um método tradicional para a coloração do biofilme.^(^^8^^,^^11^^,^^14^^)^ Contudo, agentes evidenciadores podem causar manchas extrínsecas em materiais restauradoras e protéticas, como resina acrílica, resinas compostas e ionômeros de resina modificada, causando prejuízo estético e insatisfação do paciente.^(^^16^^,^^17^^)^ Assim, desde que esses materiais são frequentemente usados na prótese sobre implante ou em restaurações de orifício de acesso ao parafuso, o uso de agentes evidenciadores é um problema. Ademais, os agentes evidenciadores foram descritos como potencialmente alergênicos.^(^^14^^)^

O uso de fluorescência tem sido muito estudado para visualização do biofilme oral relacionado a doenças periodontais e cáries.^(^^11^^–^^13^^,^^18^^)^ Nesse sentido, a técnica de fluorescência quantitativa induzida pela luz (QLF - *quantitative light-induced fluorescence*), que utiliza luz com 405nm de comprimento de onda para iluminação da cavidade oral, permite a observação de biofilme oral maduro, com coloração alaranjada ou avermelhada, em função da presença de porfirinas produzidas por certas bactérias *Gram*-negativas.^(^^8^^,^^15^^,^^19^^–^^21^^)^ Porém, mesmo com a correlação entre biofilme patogênico maduro e visualização avermelhada por fluo­rescência, o uso de QLF na rotina clínica ainda não apresenta boa relação custo-efetividade. Por outro lado, a técnica da fluorescência óptica de campo amplo (WOF - *wide-field optical fluorescence*) tem melhor relação custo-efetividade para uso na rotina clínica e aplica os mesmos princípios que QLF, devido ao uso de luz com a mesma faixa de comprimento de onda. Entretanto, até então, quase todos os estudos sobre WOF foram relacionados à detecção de lesões orais potencialmente malignas ou câncer de boca.^(^^22^^–^^24^^)^ Recentemente, demonstramos o uso de WOF na rotina clínica em diferentes áreas da odontologia e da medicina.^(^^25^^–^^27^^)^ Porém, até então, não havia relatos na literatura sobre o uso de WOF na rotina clínica, para a visualização do biofilme oral associado aos implantes.

O presente relato descreve o uso de WOF para a visualização de biofilme oral associado a implantes e dentes em uma consulta de rotina de paciente parcialmente edêntula, com implantes, que apresentava mucosite peri-implantar.

O protocolo de cuidados incluía exame clínico convencional, exame WOF, seguidos por limpeza e polimento mecânicos e instruções de higiene oral para a paciente. Uma câmera intraoral modelo DP6 Scope^®^ (RF System Lab., Almere, Holanda), acoplada a um computador, foi usada para capturar imagens clínicas. O aparelho Evince^®^ (MM Optics, São Carlos, Brasil), com a fonte de luz emitindo a 400±10nm, foi usado para a realização do WOF, enquanto microscópio digital modelo Deluxe Handheld Digital Microscope^®^ (Celestron LLC, Torrance, CA) foi usado acoplado ao Evince^®^ e a um computador, para obter a imagem por fluorescência óptica.

Os procedimentos manuais de limpeza e polimento para a remoção de biofilme oral e cálculo na região dos implantes foram feitos usando taças e cones de silicone (Microdont, São Paulo, SP, Brasil) com pasta de poli­mento (Clinpro™ Prophy Paste, 3M Oral Care, Saint Paul, MN, Estados Unidos) e um *kit* para raspagem de implantes (Implacare™, Hu-Friedy Mfg. Co., Chicago, IL, Estados Unidos). Na região dental, foi usado o ultrassom (PROFI II Ceramic, Dabi Atlante, Ribeirão Preto, SP, Brasil) com pontas de metal (Dabi Atlante, Ribeirão Preto, SP, Brasil) e raspadores metálicos (Hu-Friedy Mfg. Co., Chicago, IL, Estados Unidos). Além disso, os mesmos tipos de pasta de polimento, taças e cones de silicone usados nos implantes foram utilizados nos dentes.

Foram dadas instruções de higiene oral à paciente, apresentando as imagens de fluorescência avermelhada de biofilme oral, de modo que ela pudesse identificar regiões com o maior acúmulo e removê-lo. O protocolo de cuidados foi realizado no dia da consulta inicial e repetido após 15 dias. Trinta dias depois da primeira consulta, foram feitos somente novo exame clínico e fluorescência de campo amplo.

Este estudo foi aprovado pelo Comitê de Ética da Universidade Federal de São João del-Rei, *Campus* Centro-Oeste Dona Lindu (número de aprovação: 1.756.617; CAAE: 59621516.8.0000.5545). Em adição, declara-se que o paciente assinou o Termo de Consentimento Livre e Esclarecido.

## RELATO DE CASO

Paciente de 55 anos, do sexo feminino, parcialmente desdentada e reabilitada com próteses sobre implantes, compareceu ao Centro de Diagnóstico Oral do Departamento de Saúde de Divinópolis, em Minas Gerais, para consulta de rotina, com queixa de inchaço e sangramento na mucosa peri-implantar de implantes na região dos dentes 12 e 11, percebidos 3 meses antes. A paciente informou ter diabetes e história de diversas cirurgias por doença periodontal nos dentes posteriores, tendo feito reabilitação por meio de implantes 8 anos antes com outro profissional. Atualmente, estava sem queixa de dor.

### Observações clínicas

A presença de cárie foi verificada no dente 13, na mesial das superfícies vestibular e palatina (Figuras 1A e 1B), área edematosa e eritematosa na mucosa peri-implantar dos dentes 12 a 23, associada a cálculo dental no dente 12 e a sangramento nos dentes 12 e 11 (Figuras 1C e 1D). Por sua vez, as restaurações de resina na região do orifício de acesso do parafuso de implantes em 12,11 e 21 estavam seladas de forma adequada (Figura 1E). Foram notadas regiões eritematosas na mucosa na região vestibular do dente 25 e na região interproximal da superfície palatina da prótese, sobre implantes entre 23 e 24 (Figuras 1F e 1G). Além disso, foi identificada ausência da coroa protética associada com o implante no dente 26 (Figuras 1G e 1H). O arco inferior, por sua vez, apresentava próteses sobre implantes dos dentes 44 a 46 e dentes 34 a 43 com restaurações extensas, sem acabamento e polimento (Figuras 1I, 1J, 1K e 1L). A paciente ainda apresentava áreas edêntulas sem rea­bilitação, de 15 a 18, 27, 28, de 38 a 35, 47 e 48.

**Figura 1:** Imagens clínicas iniciais. Cárie no dente 13 sobre as superfícies mésio-bucal (A) e palatina (B); Edema da mucosa peri-implantar na região de 12 a 23, com cálculo dentário em 12 e sangramento em 12 e 11 (C e D); Restaurações de orifícios de acesso do parafuso dos implantes em 12, 11 e 21 (E); Próteses sobre implantes de 23 a 25, nos quais se observa eritema da mucosa em 25 (F); Região palatina de 24 a 26, na qual se notam eritema na face mesial de 24 e componente protético do implante na região de 26, com perda da coroa protética (G e H); Vista bucal dos implantes e suas restaurações de orifício de acesso de parafuso de 44 a 46 (I); Dentes 32 a 42 com restaurações inacabadas e sem polimento (J); Visão bucal dos dentes 33 e 34 (K) e lingual (L). O dente 34 tem restauração protética

Na sondagem, os dentes apresentaram valores ≤2mm, exceto o dente 14, que apresentava sondagem distal de 3mm, sem evidência de sangramento ou supuração, mas com discreta mobilidade. Por sua vez, os implantes apresentaram valores de sondagem de ≤3mm, com presença de sangramento apenas em 12 e 11.

### Visualização com fluorescência óptica de campo amplo

Uma fluorescência avermelhada pôde ser vista na região da cárie no dente 13, nas superfícies vestibular e palatina (Figuras 2A e 2B) e ao longo da área cervical da superfície vestibular das próteses, sobre os implantes 12 a 21, a qual foi mais intensa em 12 e 11 (Figuras 2C e 2D). Notou-se fluorescência verde intensa na região das restaurações dos orifícios de acesso dos parafusos dos implantes 12 a 21 (Figura 2E). Por sua vez, a fluorescência avermelhada foi observada na área cervical da superfície bucal de 23 a 26, no orifício de acesso do parafuso do implante em 26 e na região palatina interproximal entre 23 e 24 (Figuras 2F, 2G e 2H). O arco inferior mostrou fluorescência avermelhada na área cervical das próteses sobre implantes de 44 a 46, área cervical dos dentes de 43 a 31, interproximal entre os dentes 32 a 34 e cervical da face lingual de 34 (Figuras 2I, 2J, 2K e 2L).

**Figura 2:** Imagens iniciais da fluorescência óptica de campo amplo. Aspecto vestibular da região de 14 e 13 mostrando fluorescência avermelhada na região mésio­­bucal do dente 13 (A); Aspecto palatino da região dos dentes 13 a 21 mostrando fluorescência avermelhada no dente 13 e áreas de fluorescência verde intensa na região de restaurações dos orifícios de acesso do parafuso dos implantes 12, 11 e 21 (B); Aspecto vestibular das próteses sobre implantes de 12 a 22, mostrando fluorescência avermelhada na cervical, que é mais intensa em 12 e 11 (C e D); Aspecto palatino com fluorescência verde intensa na região das restaurações dos orifícios de acesso do parafuso dos implantes de 12 a 21 (E); Presença de fluorescência avermelhada na cervical da área bucal das próteses sobre implantes de 23 a 25 (F); Área palatina interproximal entre 23 e 24, face cervical e no acesso ao parafuso do componente protético 26 (G e H); Fluorescência verde intensa de restaurações dos orifícios de acesso aos parafusos dos implantes em 23 e 24 (F e G); Fluorescência avermelhada na região cervical das próteses sobre implantes de 46 a 44 (I); Área cervical dos dentes 43 a 31 (I e J); Interproximal entre os dentes 32 a 34 na região bucal (K) e na área cervical da face lingual do 34 (L)

### Exame radiográfico

Radiografia periapical mostrou perda óssea na porção distal do dente 14 e uma cárie na região mesial do dente 13, o qual era tratado endodonticamente (Figura 3A). Implantes dentários estavam presentes na região de 12, 11, 21, 23, 24, 26, 44, 45 e 46 com imagens dentro do padrão ósseo normal (Figuras 3A a 3E). Existiam próteses sobre implantes de 12 a 25, 46, 45 e 44, sendo que, em 26, não havia coroa protética relacionada ao implante (Figuras 3A a 3E). Por sua vez, as figuras 3F a 3H mostram perda óssea que variava de leve a moderada, relacionada aos dentes de 33 a 43, e presença de dentes restaurados, de 34 a 43. O dente 34 era tratado endodonticamente.

**Figura 3:** Imagens radiográficas iniciais. Presença de prótese sobre implante na região do dente 12, perda óssea distal do dente 14 e área radiolúcida na face mesial do dente 13, o qual era tratado endodonticamente (A); Presença de próteses sobre implantes de 12 a 25 e de 44 a 46, com padrão ósseo normal (B, C, D e E); Implante associado com seu componente protético na região do dente 26, mas com perda da coroa protética (D); Dentes 34 a 43 extensivamente restaurados (F, G e H), e 34 era tratado endodonticamente e restaurado com coroa protética (H)

### Correlações entre consultas: inicial e de acompanhamento com 15 e 30 dias

O processo de recuperação da saúde da mucosa peri-im­plantar em 12 e 11 pôde ser notado pela redução de edema e pela ausência de sangramento 15 dias após a consulta inicial (Figura 4A). Após 30 dias, a mucosa peri-implantar estava em condição normal (Figura 4B). Na consulta inicial, a região cervical de 11 e 12 mostrou a presença de fluorescência avermelhada intensa (Figura 4C), que, após 30 dias, estava ausente (Figura 4D). A região do implante correspondente ao dente 26, no qual não havia coroa protética, mostrou características de mucosa normal aos 15 (Figura 4E) e 30 dias (Figura 4F). De fato, na consulta inicial, havia fluorescência avermelhada ao redor do componente protético e no orifício de acesso do parafuso do dente 26 (Figura 4G), que, após 30 dias, estava ausente (Figura 4H). Fora isso, a presença inicial de restaurações inacabadas e não polidas na região de 32 a 42 (Figura 4I) que, após 30 dias, estavam devidamente terminadas, polidas e com a remoção do biofilme, permitiu a identificação da cárie cervical no dente 41 (Figura 4J). Entretanto, neste caso, a fluorescência avermelhada inicial na região cervical de 31, 41, 42 e 43 (Figura 4K) estava mantida após 30 dias apenas na região da cárie cervical do dente 41 (Figura 4L).

**Figura 4:** Imagens clínicas e de fluorescência referentes às consultas inicial e de acompanhamento com 15 e 30 dias. Redução do edema e ausência de sangramento da mucosa peri-implantar em 12 e 11, 15 dias após a consulta inicial (A); 30 dias após a consulta inicial, a região tinha características normais (B); Presença de fluorescência avermelhada na cervical de próteses sobre implantes de 12 a 21, que era mais intensa em 12 e 11 na consulta inicial (C); 30 dias após a consulta inicial, a mesma região mostrava ausência de fluorescência avermelhada (D); A mucosa peri-implantar na região de 26 com 15 dias (E) e 30 dias (F) após a consulta inicial, com características normais. Aspecto inicial de fluorescência avermelhada ao redor do componente protético e orifício de acesso do parafuso do implante 26 (G); 30 dias após a consulta inicial, a mesma região mostrou ausência de fluorescência avermelhada (H); Dentes 32 a 42 com restaurações inacabadas e sem polimento após 15 dias (I) e, em 30 dias após a consulta inicial, as restaurações estavam acabadas, polidas, de forma adequada, e havia presença de cárie cervical em 41 (J); Presença na consulta inicial de fluorescência avermelhada na cervical de 31 a 43 e interproximal entre 31 e 41 (K); Após 30 dias, havia fluorescência avermelhada apenas na cervical de 41 (L)

Foi observada correlação direta entre presença de fluorescência avermelhada e envolvimento da mucosa peri-implantar. Ademais, a área com o pior quadro clínico, com sangramento entre 12 e 11, correspondeu à área com a maior intensidade de fluorescência avermelhada. Foi evidente que, após a remoção adequada do cálculo e do biofilme oral, guiada pela identificação prévia de fluorescência avermelhada e acompanhada de orientação de higiene oral para a paciente, houve completo restabelecimento da saúde peri-implantar em 30 dias. O exame WOF também permitiu avaliar a integridade do selamento proporcionado pelas restaurações do orifício de acesso ao parafuso dos implantes, em função do contraste entre o material componente da prótese e o material de restauração.

O diagnóstico foi estabelecido como mucosite peri-implantar, devido ao envolvimento de apenas o tecido da mucosa peri-implantar, indolor, sem perda óssea ou supuração, e sem mobilidade em qualquer implante. Assim, a paciente foi informada da correlação entre acúmulo de biofilme oral e mucosite peri-implantar e, assim, da necessidade de manter higiene oral rigorosa, especialmente em áreas que demonstravam a fluorescência avermelhada. Também foi explicada à paciente a importância da manutenção de controle do diabetes, não apenas para prevenir a piora de sua saúde dental, mas igualmente para manter boa saúde geral. Em seguida, a paciente foi encaminhada a outro dentista, para realizar procedimentos periodontais, restauradores e protéticos e para acompanhamento de higiene periodontal e oral.

## DISCUSSÃO

Inicialmente, é importante informar que o dispositivo usado neste caso funciona com emissão de luz em 400±10nm. Assim, ele se beneficia das correlações obtidas de estudos feitos com 400 ou 405nm, já que compartilham das mesmas características biofotônicas. Portanto, a presença inicial de fluorescência avermelhada no dente 13 e após 30 dias da consulta inicial na lesão na região cervical do dente 41 é corroborada pelos achados de König et al.,^(^^28^^)^ indicando que o comprimento de onda mais adequado para a estimulação e a visualização da fluorescência avermelhada na cárie é 400nm.^(^^28^^)^ Caso contrário, a lesão cervical do dente 31 provavelmente tem origem diferente da cárie, como perda de material de restauração, e, devido à ausência de contaminação bacteriana, a fluorescência avermelhada estava ausente.

Porém, este caso pode ser considerado bem representativo das possibilidades do uso de WOF no acompanhamento de pacientes reabilitados por implantes, já que a paciente estava parcialmente edêntula e reabilitada com implantes, o que ilustra particularidades que se aplicam aos dentes e aos implantes. Neste sentido, Lee et al.,^(^^10^^)^ Quirynen et al.,^(^^3^^)^ Kurtzman et al.,^(^^9^^)^ e Todescan et al.,^(^^6^^)^ enfatizaram que deve ser tomado cuidado especial com pacientes parcialmente edêntulos, em que os dentes remanescentes funcionam como reservatórios de bactérias para colonização do implante.^(^^3^^,^^6^^,^^9^^,^^10^^)^

Estudos conduzidos com QLF por Coulthwaite et al.,^(^^19^^)^ Volgenant et al.,^(^^20^^)^ Han et al.,^(^^21^^)^ Kim et al.,^(^^15^^)^ Pretty et al.,^(^^11^^)^ e Lee et al.,^(^^8^^)^ mostraram que apenas o biofilme oral maduro revelou fluorescência em tons avermelhados ou alaranjados, o que foi possivelmente devido a bactérias anaeróbicas *Gram*-negativas.^(^^8^^,^^11^^,^^15^^,^^19^^–^^21^^)^ Além disso, Han et al.,^(^^21^^)^ enfatizaram a correlação entre fluorescência avermelhada e a presença de *Porphyromonas gingivalis, Prevotella intermedia* e *Fusobacterium nucleatum*, bactérias anaeróbicas patogênicas presentes no biofilme maduro relacionadas à doença periodontal.^(^^21^^)^ Bjurshammar et al.,^(^^29^^)^ demonstraram que *Aggregatibacter actinomycetemcomitans* é capaz de emitir fluorescência avermelhada, e há correlação entre o tempo de crescimento da colônia e o aumento da intensidade da fluorescência avermelhada.^(^^29^^)^ Assim, esses estudos anteriores validam o uso do WOF apresentado neste caso, a fim de visualizar biofilme maduro com maior potencial patogênico, permitindo sua remoção completa. Ademais, o relato da paciente sobre o tempo de evolução de 3 meses para a percepção dos sinais de mucosite peri-implantar é mais do que suficiente para estabelecer a presença de biofilme maduro e patogênico. De igual forma, a remoção do biofilme oral e a manutenção de higiene oral adequada preveniram a formação do biofilme e permitiram a recuperação da saúde da mucosa peri-implantar, que mostrou correlação com a ausência de fluorescência avermelhada 30 dias após a consulta inicial.

A verificação do selamento fornecido pelas restaurações de acessos aos parafusos dos implantes é de fundamental importância para evitar que, no caso de uma falha, seja permitida a retenção ou penetração de biofilme nessas áreas. Nesse sentido, Heinrich-Weltzien et al.,^(^^18^^)^ destacaram que a presença de fluorescência avermelhada pode indicar penetração do biofilme oral na restauração, microinfiltração, lacuna ou perda parcial da restauração.^(^^18^^)^ Assim, neste relato de caso, o contraste entre o material de restauração com fluorescência de cor verde clara brilhante e o material da prótese, com fluorescência mais baixa, foi prontamente verificado por WOF. Portanto, as restaurações para selamento do acesso dos parafusos foram consideradas adequadas devido à não visualização da fluorescência avermelhada.

Heinrich-Weltzien et al.,^(^^18^^)^ relataram que a fluores­cência assegura a prática de remoção do biofilme oral por meio do direcionamento de sítios específicos e, também, permite ao profissional garantir que todo o biofilme tenha sido removido após a limpeza profissional.^(^^18^^)^ Da mesma forma, neste relato de caso, o uso de fluorescência de campo amplo permitiu a visualização do biofilme oral maduro em tons avermelhados, que serve para direcionar tanto o profissional quanto o paciente relativo a quais áreas devem receber mais atenção na remoção do biofilme oral.

## CONCLUSÃO

A fluorescência óptica de campo amplo pode ser usada na rotina clínica de pacientes com implantes dentários. Ela permitiu visualização fácil e imediata do biofilme oral maduro, para sua remoção adequada, avaliação da qualidade da restauração para selamento do orifício de acesso do parafuso e identificação de cáries em pacientes parcialmente edêntulos, sem o risco de reações alérgicas ou manchamento de próteses e restaurações.
